# The Endocannabinoid System in the Development and Treatment of Obesity: Searching for New Ideas

**DOI:** 10.3390/ijms26199549

**Published:** 2025-09-30

**Authors:** Anna Serefko, Joanna Lachowicz-Radulska, Monika Elżbieta Jach, Katarzyna Świąder, Aleksandra Szopa

**Affiliations:** 1 Department of Clinical Pharmacy and Pharmaceutical Care, Medical University of Lublin, 1 Chodźki Street, PL 20-093 Lublin, Poland; joanna.lachowicz@umlub.pl (J.L.-R.); aleksandra.szopa@umlub.pl (A.S.); 2 Department of Molecular Biology, The John Paul II Catholic University of Lublin, Konstantynów Street 1I, PL 20-708 Lublin, Poland; monijach@kul.pl; 3 Chair and Department of Applied and Social Pharmacy, Medical University of Lublin, 1 Chodźki Street, PL 20-093 Lublin, Poland; katarzyna.swiader@umlub.pl

**Keywords:** endocannabinoid system, obesity, cannabinoid-based therapy, CB1 receptor, CB2 receptor

## Abstract

Obesity is a complex, multifactorial disease and a growing global health challenge associated with type 2 diabetes, cardiovascular disorders, cancer, and reduced quality of life. The existing pharmacological therapies are characterized by their limited number and efficacy, and safety concerns further restrict their utilization. This review synthesizes extensive knowledge regarding the role of the endocannabinoid system (ECS) in the pathogenesis of obesity, as well as its potential as a therapeutic target. A thorough evaluation of preclinical and clinical data concerning endocannabinoid ligands, cannabinoid receptors (CB1, CB2), their genetic variants, and pharmacological interventions targeting the ECS was conducted. Literature data suggests that the overactivation of the ECS may play a role in the pathophysiology of excessive food intake, dysregulated energy balance, adiposity, and metabolic disturbances. The pharmacological modulation of ECS components, by means of CB1 receptor antagonists/inverse agonists, CB2 receptor agonists, enzyme inhibitors, and hybrid or allosteric ligands, has demonstrated promising anti-obesity effects in animal models. However, the translation of these findings into clinical practice remains challenging due to safety concerns, particularly neuropsychiatric adverse events. The development of novel strategies, including peripherally restricted compounds, hybrid dual-target agents, dietary modulation of endocannabinoid tone, and non-pharmacological interventions, promises to advance the field of obesity management.

## 1. Introduction

According to the Human Phenotype Ontology (HPO), increased body weight, which is classified within the abnormalities of body weight, is divided into three phenotypes, i.e., large for gestational age, obesity, and overweight (Table 1). As indicated by the World Health Organization, there were 890 mln adult people with obesity in 2022, which corresponds to 1 in 8 people worldwide. The prevalence of the obesity epidemic is still rising around the world, regardless of sex and age. It has been estimated that without a wide-ranging intervention, almost two in three adults over the age of 25 will be obese/overweight [1]. Generally, obesity develops due to an imbalance between energy intake (food consumption) and expenditure (physical activity), which depends on several factors, i.e., environmental, psycho-social, behavioral, economic, and genetic factors. Obesity is not only an esthetical problem. It increases the risk of type 2 diabetes, heart disease, and certain cancers. In addition, it can have a negative impact on reproduction, sleep, bones, and movement. Basically, obesity significantly worsens the quality of life [2].

Lifestyle changes, including proper diet and physical activity, which are inseparable, are the first non-pharmacological methods used in the management of obesity. Currently, only a few drugs are registered for the treatment of obesity, i.e., orlistat, naltrexone hydrochloride plus bupropion hydrochloride, phentermine plus topiramate, liraglutide, semaglutide, and tirzepatide. Several compounds were withdrawn from the pharmaceutical market due to safety concerns, including fenfluramine, dexfenfluramine, sibutramine, and lorcaserin. Insufficient efficacy with bothersome side effects are two main issues related to the medications used in weight reduction. Research on novel drug targets and active substances has been carried out. Of course, most of this research consists of laboratory and preclinical studies, but there are several new anti-obesity drugs at the final stage of their development, in clinical trials, e.g., danuglipron, survodutide, mazdutide, retatrutide, cagrilintide. Most of the above-mentioned agents act via the agonism of the glucagon-like peptide-1, glucagon, and/or glucose-dependent insulinotropic polypeptide receptors [2]. Nevertheless, a promising new compound is also monlunabant, an orally bioavailable inhibitor of cannabinoid CB1 receptors. An estimated date of its approval in the US is 2028 at the earliest [3].

Energy status (monitored via hormones and nutrients), pleasure of eating, food reward, and learned associations with eating (e.g., a dressed table) are responsible for feeding behavior. Numerous neuronal circuits that are modulated/controlled by dopaminergic, serotonergic, gamma-aminobutyric acid (GABA)-ergic, and opioid pathways are involved in this complex process. In vivo studies and clinical trials have proven that the endocannabinoid system (ECS) has a wide impact on both the physiology and metabolism of multiple systems within a living organism, including the brain, liver, bones, muscles, gastrointestinal system, immune system, and adipose tissues. It plays an important role in motor and immune functions, thermoregulation, nociception, neuropathic pain, memory, cognition, sleep, and stress and reward responses [4]. On a molecular basis, the ECS is implicated in several fundamental processes in the central nervous system (CNS), such as synaptic plasticity, neural development, and inflammatory responses [5,6]. Endocannabinoids are able to induce short-term changes in synaptic transmission and longer-term plasticity at both inhibitory and excitatory synapses. They can transiently (for ≤1 min) suppress synaptic transmission, which is caused either by short-term depression (STD) or by depolarization-induced suppression of inhibition (DSI)/excitation (DSE). This process, mediated by endocannabinoids, is observed in different brain areas, including the hippocampus and cerebellum, and it can modulate local neuronal activity related to reward circuits. Endocannabinoids also produce long-term changes in synaptic transmission (lasting for hours–weeks), known as long-term depression (LTD) and long-term potentiation (LTP). Endocannabinoid-mediated LTD is the better-described type. It seems to be implicated in the learning and addiction-related behaviors (in the prefrontal cortex–striatal area), proper control of movements (in the corticostriatal area), and sensory processing (in the somatosensory cortex, visual cortex, and cerebellar cortex). As for endocannabinoid-mediated LTP, this type of plasticity may have an impact on associative learning tasks (for a review, see [7]). The activity of the ECS has been observed since the early stages of neural tissue formation. Endocannabinoid-dependent signaling affects various neurogenic processes, from neuronal proliferation, specification, and maturation to the maintenance and survival of differentiated neural cells. Furthermore, cannabinoid CB1 receptors take part in the regulation of neuritogenesis, synaptogenesis, and neuronal migration [6,8]. It has also been shown that astrocytes and microglial cells produce endocannabinoids, and there is an endocannabinoid-mediated communication between microglia and neurons [5]. The ECS is highly important in physiological responses to brain trauma, neurological inflammation, and neurodegenerative conditions. Although a particular role is assigned to cannabinoid CB2 receptors that inhibit neuroinflammatory signaling pathways and change glial activity from a pro-inflammatory state to an anti-inflammatory one [6,9], neuroprotection against excitotoxicity, mediated by CB1 receptors via inhibitory effects on Ca^2+^ influx, glutamate release, production of nitric oxide, and zinc mobilization with the indirect potentiation of brain-derived neurotrophic factor (BDNF) expression, has been demonstrated as well [5].

Preclinical data and data from human studies indicate that the endocannabinoid transmission also plays a crucial role in food intake and energy balance; it controls processes related to food seeking, eating, metabolism, and calorie preservation; and it facilitates motivation towards eating. These effects are highly positive in the case of limited food availability but may be unhealthy when food is easily accessible, i.e., they may lead to the development of obesity. Endocannabinoids increase appetite, and since they take part in the modulation of taste and smell, they enhance sweet taste sensitivity, influence fat taste perception, and promote the consumption of palatable food, reinforcing its rewarding effects [10,11,12,13,14]. It was even suggested that the ECS potentiates the effect of food palatability; thus, it increases its hedonic value [15]. The ECS also contributes to energy storage by stimulating peripheral anabolic processes. Enhanced motivation-directed behavior for food and an increased food hedonic effect, both induced by endocannabinoids, are dependent on dopamine activity. Additionally, the nutritional status and diet affect the ECS both transiently and in a long-lasting manner. Feeding and fasting induce changes in endocannabinoid levels in the CNS and in the periphery, which was observed in experiments carried out in animals. Fasting elevates the brain levels of endocannabinoids, particularly within the hypothalamus (responsible for homeostatic feeding) and limbic forebrain (responsible for hedonic feeding), whereas feeding may reduce these levels [16,17,18]. In humans, it was detected that exposure to food alters endocannabinoid levels in plasma, which may be influenced by the palatability of food and lean/obesity status [19,20]. The first data on the pro-appetizing effect of cannabis in humans come from 300 AD. Clinical evidence on the weight-increasing effect of cannabis is scarce, but dronabinol, a synthetic form of delta-9-tetrahydro-cannabinol, serves as a well-tolerated option for appetite stimulation in HIV/AIDS patients [21]. As mentioned above, various dietary interventions have an impact on endocannabinoid levels. It was demonstrated that a diet rich in fat and/or sucrose is able to increase levels of endocannabinoids and change the expression of both cannabinoid receptors and enzymes engaged in the biosynthesis/degradation of endocannabinoids [22,23]. The ingestion of dietary fatty acids (e.g., omega-3 and omega-6 polyunsaturated fatty acids) that are precursors of endocannabinoids may affect their levels [24,25,26]. The outcomes of a parallel 8-week randomized controlled trial with the participation of obese healthy people demonstrated that the isocaloric shift from a Western diet to the Mediterranean diet reduced the plasma levels of arachidonoylethanolamide (*N*-arachidonoyl-ethanolamine, anandamide, AEA) [27]. The Mediterranean diet contains cannabinomimetic molecules that mainly belong to polyphenols and terpenes, e.g., β-caryophyllene, which in the nanomolar range acts as a selective agonist of CB2 receptors, improves glucose and insulin parameters, as well as ameliorates the lipid profile [28]; resveratrol modulates different components of the ECS [29]; and kaempferol and biochanin A inhibit fatty acid amide hydrolase [29,30,31], whereas quercetin [32,33] and extra-virgin olive oil [34,35] seem to upregulate the expression of CB1 receptors [32,33,36].

Along with the CNS, the peripheral nervous system is essential in feeding behavior, metabolism, and digestion. Information from the periphery is conveyed to the CNS. Receptors for endocannabinoids are also expressed peripherally, in the adipose tissue, liver, skeletal muscle, pancreas, kidney, and gastrointestinal tract. The ECS has an impact on gastrointestinal contractility and secretion, since cannabinoid receptors can be found on vagal afferent and efferent fibers and the sensory neurons of the enteric nervous system [37]. Apart from that, the ECS plays a crucial role in the gut–brain communication. When overactivated in the intestine (i.e., in obesity), it exerts changes in gut microbiota and gut permeability [38,39]. It was shown that endocannabinoids interplay with neurotransmitters or hormones that regulate hunger/satiety, including leptin, ghrelin, orexin, cholecystokinin, glucagon-like peptide 1, neuropeptide Y, and others [40,41].

The outcomes of many studies, both in animals and with the participation of human subjects, have given evidence that the overactivation of the ECS contributes to the development of obesity. For example, (1) An increased expression level of cannabinoid CB1 receptors is observed in obesity [42,43,44]. (2) The stimulation of CB1 receptors in the brain leads to an increased appetite, intensified reward aspects of eating, and a higher level of food intake, which promotes lipogenesis, adipogenesis, the accumulation of fat, and the impairment of glucose uptake, and as a consequence, it results in an elevated body weight [43,44]. (3) The plasma levels of endocannabinoids correlate with obesity markers [42,45], including body mass index, waist circumference, insulin resistance, and visceral fat mass [19,46,47,48,49,50,51,52], as well as with some genetic causes of obesity [53]; in a study carried out by Fanelli and colleagues (2018) [54] AEA hypertone was independent of gender, age, or fertility status. (4) Variations in genes encoding cannabinoid receptors, fatty acid amide hydrolase, or *N*-acyl phosphatidylethanolamine phospholipase D are detected in overweight/obese people [55,56,57,58,59], which may influence food preference and eating behavior [60,61,62]. (5) South Asians that have an elevated risk of developing obesity present a higher endocannabinoid tone [63]. (6) The dysregulation of the ECS may result in hedonic overeating, excessive fat storage, and body mass gain [64]. Contrarily, the inhibition of CB1 receptors results in decreased food intake, weight loss, and improved lipid metabolism and sensitivity to insulin [65,66]. However, it should be emphasized that the activation of CB1 receptors in the brain not always results in appetite-inducing responses—the effect is often dependent on their location. Anorectic activity is associated with the inhibition of GABAergic transmission, whereas orexigenic activity is mediated via glutamatergic regulation [67]. Interestingly, the obesogenic effects of some organophosphate pesticides [68] or bisphenols [69] are due to the overstimulation of the ECS. Recent studies carried out by Calvino et al. (2024) [70] demonstrated that maternal obesity is able to induce specific sex-dependent molecular modifications in the ECS (increased expression of CB1 receptors with decreased expression of CB2 receptors in the hypothalamus as well as reduced level of monoacylglycerol lipase) of the offspring that predispose them to the development of metabolic disturbances. These findings were in accordance with previous reports by Fassarella et al. (2024) [71] and Dias-Rocha and colleagues (2018) [72] related to the association of the maternal high-fat diet with sex-specific changes in the ECS of newborn rats (i.e., elevated content of CB1 receptors in the hypothalamus and liver along with decreased levels of CB1 receptors and increased levels of fatty acid amide hydrolase in the brown adipose tissue in male pups versus elevated content of CB2 receptors in the hypothalamus and liver along with increased levels of CB2 receptors and monoacylglycerol lipase in female pups). Furthermore, both sexes at adult age presented a higher mass of the white adipose tissue along with increased body weight and food intake (with a preference for the high-fat diet). The preference for fat was also detected in male adolescent offspring [73]. Similar outcomes were obtained by Miranda et al. (2018) [74] who noted that a maternal high-fat diet results in elevated body weight and the overactivation of the ECS in adult offspring, particularly in male progeny (higher levels of CB1 and CB2 receptors as well as increased content of fatty acid amid hydrolase and monoacylglycerol lipase in the liver). Since the ECS takes part in hunger/satiety control, agents that can alter the activity of the ECS have gained interest as new therapeutic perspectives in the management of obesity. The modulation of cannabinoid receptors (CB1 and CB2) and inhibition of fatty acid amide hydrolase or monoacylglycerol lipase are predominantly tested.

The main objective of this review is to highlight recent advances in research on the role of the ECS in the development and treatment of obesity. Because this topic is very broad, we decided to focus on conventional endocannabinoids (i.e., anandamide and 2-arachidonoylglycerol) and their main receptors (CB1 and CB2), even though endocannabinoid-like compounds (such as palmitoylethanolamide, *N*-acylethanolamines, oleylethanolamide, and 2-monoacylglycerols) and several cannabinoid-related receptors, such as G protein-coupled receptor 55 (GPR55), G protein-coupled receptor 119 (GPR119), and transient receptor potential vanilloid 1 receptor (TRPV1R), are involved in feeding behavior and energy expenditure [75,76,77,78]. This review will discuss the association of feeding behavior with the ECS, consequences of genetic variants within the ECS for food intake and body weight, and cannabinoid-based treatments in the management of obesity with new perspectives in this field.

## 2. The Endocannabinoid System

The ECS is a well-conserved system that consists of nine endogenous ligands, which are derived from fatty acids, i.e., AEA, 2-arachidonoylglycerol (2-AG), *N*-eicosapentaenoylethanolamide, *N*-docosahexaenoyl-ethanolamide [79], enzymes involved in their formation and breakdown, and receptors for endocannabinoids, i.e., CB1 and CB2 receptors. They belong to the G protein-coupled receptor family. The structure of both cannabinoid receptors aligns with the canonical model of class A G protein-coupled receptors (GPRs), featuring a glycosylated extracellular amino-terminal (N-term) domain and an intracellular carboxyl-terminal (C-term) domain interconnected by a seven-transmembrane (7TM) helical bundle [80,81]. The 7TM core is linked by three extracellular loops (ECL1–3), which shape the ligand-binding vestibule, and three intracellular loops (ICL1–3), which participate in G protein and arrestin coupling [82,83,84,85]. CB1 and CB2 receptors exhibit approximately 44% of the overall amino acid sequence identity [80,81,83,86,87], with markedly higher conservation (~68%) across the 7TM helical domains that form the structural core of the receptors [87,88,89]. Conversely, the N-term segment, ECL2, and the intracellular C-term domain exhibit the most significant sequence divergence. These regions have been demonstrated to contribute to subtype-specific differences in ligand recognition, receptor trafficking, and intracellular signaling bias [83,88,90]. Structural analyses of crystallographic and cryo-EM models (e.g., CB1: PDB code 5TGZ, 6N4B; CB2: PDB code 5ZTY, 6PT0) [91,92,93,94] confirm that, while the 7TM bundle and conserved activation motifs (DRY, NPxxY, toggle switch) are closely aligned, differences in N-term length and conformation, the architecture of ECL2, and C-term extensions underlie functional divergence between the two receptors [82,92,93]. Table 2 presents the cryo-EM structure of the human cannabinoid receptors CB1 and CB2 in complex with signaling proteins. Cannabinoid receptors primarily couple to the inhibitory Gi/o proteins that reduce the activity of adenylate cyclase and, as a result, decrease intracellular cyclic adenosine monophosphate levels. Their signal transduction is also mediated by the inhibition of voltage-gated calcium channels and protein kinase A, along with the activation of mitogen-activated protein kinases (MAPKs), potassium channels, P38, Rho kinase, and Rho-associated coiled-coil kinase (ROCK). They have an impact on synaptic plasticity, cell migration, and neuronal growth [88,95].

CB1 receptors are encoded by the *CNR1* gene and CB2 receptors by the *CNR2* gene. The expression of cannabinoid receptors is not uniform—CB1 receptors are located throughout the human body, including in the liver; reproductive, cardiovascular, and gastrointestinal systems; skeletal muscles; immune cells; vascular tissue; adipocytes; and the CNS (particularly in the prefrontal cortex, basal ganglia, hippocampus, and cerebellum). CB2 receptors are mainly situated peripherally (primarily in the immune cells and, to a lesser extent, in the gastrointestinal tract, peripheral nervous system, liver, and adipose tissue); however, they are also present in the CNS (e.g., in the hippocampus, striatum, thalamus, or ventral tegmental area). In vitro and preclinical studies demonstrated that 2-AG binds to both CB1 and CB2 receptors as their full agonist. As for AEA, it has a significantly higher affinity towards CB1 receptors than towards CB2 receptors, and it is a partial agonist of these receptors [98]. It should be highlighted that AEA and 2-AG are also able to activate other receptors, including GPR55, TRPV1, and peroxisome proliferator-activated receptor alpha (PPARα) and gamma (PPARγ) receptors [66,99,100]. AEA and 2-AG, which are the most frequently tested endocannabinoids, belong to n-6 polyunsaturated fatty acids and derive from membrane phospholipids precursors and arachidonic acid, but their biosynthesis depends on different enzymes—*N*-arachidonoyl phosphatidylethanolamine phospholipase D is the main enzyme responsible for the production of AEA, whereas diacylglycerol lipase (α and β isoforms) is the main enzyme that takes part in the formation of 2-AG [98,101,102]. These endogenous cannabinoid receptor ligands are synthesized on demand, since they are not stored [103,104,105]. Their synthesis and secretion occur at the time of intense neural activity. The brain levels of 2-AG are 170 times higher than AEA levels; however, the amount of synthesized endocannabinoids depends on multiple factors, including physiological/pathological conditions. Apart from the brain, several other tissues, like the liver, intestine, pancreas, muscles, and immune cells, are able to produce endocannabinoids. AEA and 2-AG are degraded through hydrolysis and/or oxidation. In the hydrolytic pathway, fatty acid amide hydrolase degrades both AEA and 2-AG, whereas monoacylglycerol lipase acts only on 2-AG; in the oxidative pathway, AEA and 2-AG can be broken down by cyclooxygenase-2 and different lipoxygenases [106,107,108,109]. The ECS acts through retrograde transmission in neuronal synapses, which means that endogenous cannabinoids are synthesized and released postsynaptically, they bind to presynaptic CB1 receptors, and after that, they are degraded in presynaptic neurons [110]. CB1 receptors are predominantly expressed presynaptically at GABAergic and glutamatergic terminals. Thus, their activation has an impact on GABAergic and glutamatergic neurotransmissions. It has been demonstrated that the ECS suppresses GABAergic and glutamatergic presynaptic inputs, exerting a disinhibitory and inhibitory effect, respectively, in the corresponding synapse. However, it should be noted that CB1 receptors are also expressed in serotonergic, noradrenergic, and cholinergic neurons [95]. Recent studies by Srivastava and colleagues (2022) [111] showed that mice with the deletion of CB1 receptors from catocholaminergic neurons (i.e., dopamine β-hydroxylase-expressing cells) when fed with a high-fat diet gained less body weight, displayed a higher energy expenditure, and had a better metabolic plasma profile when compared to their wild-type counterparts. Moreover, they presented increased noradrenaline turnover, expression of β-adrenergic receptors, and sympathetic tone in the visceral fat along with reduced plasma levels of corticosterone.

Generally, overactivity within the ECS may be a consequence of overexpressed cannabinoid receptors and enhanced endocannabinoid biosynthesis or their reduced degradation. Additionally, as has been demonstrated, obesity traits may also be associated with polymorphisms in genes related to the ECS, i.e., *CNR1*, *CNR2*, *NAPE-PLD* (*N*-acyl phosphatidylethanolamine phospholipase D), *FAAH1* (fatty acid amide hydrolase 1), *FAAH2* (fatty acid amide hydrolase 2), and *MGL* (monoglyceride lipase). Experiments carried out in mice with the CB1 cannabinoid receptor knockout demonstrated that these animals had a lean phenotype along with diminished plasma levels of insulin and leptin. When fed with a high-fat diet, neither developed obesity nor insulin resistance nor presented hyperphagia, which is characteristic in subjects with preserved CB1 receptors [112]. Furthermore, rodents with disrupted genes encoding CB1 receptors may exhibit a decreased level of spontaneous caloric intake [113]. Several research teams (i.e., [114,115,116]) demonstrated that even the selective deletion of the *CNR1* gene (in adipocytes, hypothalamus, or forebrain) is sufficient to obtain a lean phenotype. The reduced expression of CB1 receptors in adipocytes or the forebrain protected animals from diet-induced obesity and/or obesity-related metabolic alterations [114,116]. Furthermore, the results of genetic studies have proven the existence of *CNR1* gene variations associated with obesity and/or metabolic syndrome [55,117,118,119]. There are speculations that genetic variations in the CB1 receptor may be associated with obesity via overfeeding [120]. It was demonstrated that the single-nucleotide polymorphisms in the *CNR1* gene corresponded with weight gain and obesity: for example, rs806378 variation correlated with antipsychotic-induced weight gain in patients with European ancestry [118], rs806381 with higher body mass index in Swiss and Danish subjects [55], rs806368 with increased body mass index and waist circumference [121,122,123], and 4895 C/T with obesity in Japanese men [119]. These observations may be dependent on patients’ ethnic backgrounds, age, and sex. Some of the trials were carried out in a relatively small population of patients, and they need to be confirmed in a larger sample. Furthermore, according to Peeters et al. (2007) [120], the absence of a *CNR1* gene with the G-allele at position 1422 elevates the risk for obesity in males (but not in females), and it is related to increased abdominal adiposity in obese men. According to Benzinou and colleagues (2008) [55] or Col Araz and colleagues (2012) [58], the single-nucleotide polymorphisms of the *CNR1* gene (e.g., *CNR1* 1359G/A polymorphism) were also associated with childhood obesity. Though scientists mainly focus on the role of CB1 receptors in the regulation of feeding behavior as the receptors that play the most important role, Ishiguro and colleagues (2010) [124] demonstrated that CB2 receptors are also involved in the regulation of food intake, since polymorphism in the *CNR2* gene is associated with eating disorders. It was demonstrated that CB2 receptors may have a protective function in obesity, since their activation induces anti-inflammatory effects [125,126,127]. As a consequence, the genetic deletion of CB2 receptors results in a higher level of food intake and increased body weight versus their wild-type counterparts [126,127]. On the other hand, CB2 receptor deficiency protected the tested animals from age-related and diet-induced resistance to insulin [126]. Rossi et al. (2016) [128] paid attention to the correlation between a common missense CB2 receptor variant that reduces CB2 receptor functions, i.e., Q63R, and a high z-score body mass index in Italian children, whereas de Luis and colleagues (2017) [129] found that a single-nucleotide polymorphism rs3123554 of the *CNR2* gene was associated with body weight as well as metabolic parameters. A-allele carriers presented a higher body mass index; weight; waist circumference; fat mass; triglyceride, leptin, and insulin levels; and insulin resistance when compared to non A-allele carriers [129].

Wangensteen et al. (2011) [59], carrying out a cohort study in the Norwegian population, revealed that a common single-nucleotide polymorphism rs17605251 in the gene encoding *N*-acyl phosphatidylethanolamine phospholipase D, participating in the synthesis of AEA, is associated with obesity. Mice with a deleted *FAAH* gene presented increased body weight and enhanced motivation for food [130]. Furthermore, they had insulin resistance; reduced levels of adiponectin; high levels of triglycerides in plasma, liver, skeletal muscle, and adipose tissue; high hepatic levels of diacylglycerols; high plasma levels of free fatty acids; and elevated leptin levels and total amount of adipose tissue [130,131,132]. Even an exclusive *FAAH* knockdown in agouti-related protein neurons was sufficient to induce a similar trend in feeding behavior [133]. This is not surprising, since the disruption of fatty acid amide hydrolase activity may result in the elevation of circulating cannabinoid levels. Genetic alterations of the *FAAH* gene can lead to elevated levels of circulating endocannabinoids [134,135]. Other research teams reported that a single-nucleotide polymorphism in *FAAH* (rs324420) was associated with increased obesity [121,136,137] and may contribute to altered leptin sensitivity in humans [138], though there are discrepancies between the outcomes of different studies [137]. In their recent systemic review, based on 28 studies with 28,183 individuals, Lopez-Cortes and colleagues (2024) [137] concluded that the presence of the above-mentioned gene variant may result in a higher body mass index, fat mass, waist circumference, and waist-to-hip ratio, as well as disturbances of glucose and/or lipid homeostasis. However, the authors emphasize that these data should be interpreted with caution. The rs324420 variant contains a nucleotide missense change at position 385 from cytosine to adenine, resulting in a non-synonymous amino acid substitution from proline to threonine. There are several consequences of such a swap, including higher sensitivity to proteolytic degradation, reduced FAAH levels, and elevated AEA levels [137]. Sipe and colleagues (2005) [56] indicated the *FAAH* 385 A/A (P129T) missense polymorphism as a risk factor in overweight/obesity particularly in white and black subjects, whereas Monteleone et al. (2008) [57] and Yagin et al. (2019) [139] confirmed over-representations of the *FAAH* 385 A allele in overweight/obese in Caucasian and Iranian females, respectively. Theti and colleagues (2020) [140] found that Africans Americans have a higher prevalence of *CNR1* 3813A/G and *FAAH* C385A polymorphisms the latter when compared to Caucasians, and the latter group had a higher prevalence of the *CNR1* 4895A/G polymorphism. According to the study outcomes of Mansouri and colleagues (2020) [141], individuals with the *FAAH* genetic variant C385A not only may be prone to developing obesity, but they could also present a greater vulnerability for addiction, due to the higher expression level of dopamine D3 receptors in the brain. Interestingly, according to the latest report by Vinci et al. (2025) [142], a hemizygous variant in the *FAAH2* gene is related to metabolic disturbances, including obesity. The *FAAH2* gene, associated with the chromosome X, is widely expressed in the human body. It encodes the FAAH2 enzyme that is localized to lipid droplets and membranes and takes part in lipid catabolism, including the degradation of *N*-acylethanolamine. It should be mentioned that *FAAH2* variants may also be associated with some neuropsychiatric symptoms (e.g., anxiety or autistic features) [142].

Finally, mice with overexpressed small intestinal monoacylglycerol lipase, which were fed with a high-fat diet, presented an obese phenotype: increased weight gain, body fat mass, and hepatic and plasma levels of triglycerides; reduced energy expenditure; and hyperphagia [143]. On the other hand, transgenic rodents that overexpress monoacylglycerol lipase in forebrain neurons were lean and resistant to diet-induced obesity [144]. Animals with the global deletion of the *MGL* gene presented a leaner phenotype with an improved serum metabolic profile when compared to their wild-type counterparts [145]. They were protected from insulin resistance [146]. Mice with a double CB1 and monoacylglycerol lipase deletion had less preference for a high-fat diet, and the oral administration of lipids strongly suppressed their appetites [146].

## 3. Feeding Behavior and Its Association with the Endocannabinoid System

Feeding is a complex behavior, modulated by both central and peripheral factors dependent on nutritional status, energy expenditure, and food availability. The signals that influence food intake come from sensory organs and peripheral systems (i.e., gastrointestinal tract, liver, adipose tissue, muscles, and peripheral endocrine glands). They can be carried to the CNS with blood, or they may directly stimulate peripheral sensory terminals (e.g., afferent projections of the vagus nerve) that reach the brain areas responsible for appetite. Feeding is regulated by homeostatic (dependent on metabolic needs) and non-homeostatic (dependent on rewarding, contextual, and emotional aspects) mechanisms [147,148]. Previous experiences and/or epigenetic variations may influence these mechanisms. Thus, they may vary between individuals. The homeostatic mechanism and maintenance of energy balance are predominantly controlled by the hypothalamus and its various nuclei [149]. Orexigenic neuropeptide Y and agouti-related peptide neurons and anorexigenic pro-opiomelanocortin neurons that belong to the neurons of the arcuate nucleus are crucial to the homeostatic regulation of feeding behavior, since the first-mentioned promote feeding, whereas the others suppress it [150,151]. Neuropeptide Y/agouti-related peptide and pro-opiomelanocortin enable communication between different parts of the hypothalamus that are involved in feeding behavior and between the CNS and peripheral organs/tissues that are responsible for the consumption of food, i.e., the gastrointestinal tract, pancreas, and adipose tissue, which release ghrelin, insulin, and leptin, respectively [152]. Furthermore, the hypothalamus interplays with other brain regions, such as the nucleus of the solitary tract (conveying visceral inputs), mesolimbic reward system, and cognitive centers—the amygdala, hippocampus, and prefrontal cortex [152,153]. On the other hand, the non-homeostatic mechanism depends on brain regions involved in hedonic reactions (in this case, pleasure caused by food intake) and incentive reactions (in this case, motivation to eat). The first-mentioned are the medial shell of the nucleus accumbens, ventral pallidum, orbitofrontal cortex, posterior insula, and parabrachial nucleus, which are sensitive to opioids, orexin, and endocannabinoids. Incentive reactions are controlled by the core and shell of the nucleus accumbens, central amygdala, lateral hypothalamus, dorsolateral striatum, and prefrontal cortex, and they are predominantly regulated by dopaminergic, glutamatergic, opioid, and endocannabinoid neurotransmissions [154]. It should be mentioned that all the above-described mechanisms interplay with each other; they do not act separately [153]. Together they detect multiple neuronal, neuro-endocrine, and nutritional signals that either suppress or stimulate the need to eat. Amongst these “signals”, leptin, ghrelin, glucagon-like peptides, cholecystokinin, insulin, and glucagon play the most important role. Leptin, produced by the adipose tissue, is a hormone that reduces appetite and feeding. Ghrelin, released in the gastrointestinal tract, is a hormone that stimulates appetite and food intake. Glucagon-like peptides, produced by the intestine, inhibit appetite and give a feeling of satiety. Cholecystokinin, released from the duodenum during digestion, is a hormone that suppresses hunger. Insulin and glucagon, produced by the pancreas, work together—they regulate the levels of blood sugar and ensure a permanent supply of energy for the body. In addition to hormonal signals, gastrointestinal vagal afferents transmit information to the brain on the degree of distension of the stomach, which has an impact on the feelings of fullness and satiety [155].

Both the hypothalamus, responsible for homeostatic feeding, and mesolimbic system, responsible for non-homeostatic feeding, are abundant in CB receptors [40]. In fact, the outcomes of the study carried out by Ceccarini and colleagues (2016) [156] involving participants with food intake disorders demonstrated that CB1 receptors in the cerebral homeostatic system are associated with body mass index, and under the conditions of abnormal body weight, the brain’s reward-related areas are also implicated [156]. Zeltser et al. (2012) [157] suggested that the activity of the ECS in the hypothalamus depends on the nutritional state of the organism. It also responds to metabolic hormones, such as leptin, ghrelin, insulin, cholecystokinin, and glucocorticosteroids [158,159,160]. For example, the hypothalamic levels of endocannabinoids are positively correlated with the serum levels of orexigenic ghrelin and negatively correlated with the serum levels of anorexigenic leptin [161]. Disrupted leptin-dependent signaling results in elevated levels of endocannabinoids in the hypothalamus. Furthermore, the release and activity of fatty acid amide hydrolase are stimulated by leptin [138]. As for orexigenic glucocorticoids, they suppress the activity of hypothalamic neurons via retrograde endocannabinoid release and its inhibitory influence on glutamatergic terminals [161]. Literature data indicate that the results of the stimulation of CB1 receptors located in different parts of this brain structure may vary. In the arcuate nucleus, a dual effect of the activation of CB1 receptors was demonstrated on pro-opiomelanocortin neurons: low doses of CB1 receptor agonists lead to the depolarization of these neurons, and high doses of CB1 receptor agonists generate the hyperpolarization of these neurons. Such a phenomenon is most probably due to the fact that CB1 receptors located on pro-opiomelanocortin neurons are expressed on both GABAergic and glutamatergic presynaptic terminals, which act differently in response to the stimulation of CB1 receptors [150,151,162]. As for hypothalamic neuropeptide Y and agouti-related peptide neurons [113], it has been assumed that endocannabinoids cause their retrograde disinhibition [163], but most probably, the activation of CB1 receptors has a minor direct effect on these neurons. The modulation of the neurons of the arcuate nucleus by endocannabinoids is partly mediated by leptin, insulin, and 5′ adenosine monophosphate-activated protein kinase (AMPK) signaling. During fasting, the levels of endocannabinoids increase, which stimulates CB1 receptors and activates AMPK signaling [164,165]. Potentiated AMPK activity increases orexigenic signals either by promoting the production of neuropeptide Y or the inhibition of pathways linked to anorexigenic pro-opiomelanocortin neurons. After food consumption, when the levels of endocannabinoids drop and the activity of CB1 receptors is suppressed, the activation of hypothalamic AMPK is also much lower [165]. Endocannabinoids have a complex effect on orexin/hypocretin neurons and melanin-concentrating hormone neurons located in the lateral hypothalamus. Huang et al. (2007) [166] have shown that cannabinoids may depolarize and enhance the activity of melanin-concentrating hormone neurons (most probably by presynaptic inhibition of GABA release from nearby hypothalamic GABA neurons) but reduce the activity of hypocretin neurons (most probably by presynaptic inhibition of glutamate release). Furthermore, CB1 receptors that are present on both the excitatory and inhibitory presynaptic inputs of orexin/hypocretin neurons may act differently in lean and obese animals [167]. The stimulation of the leptin receptor in the lateral hypothalamus reduces the release of endocannabinoids and increases GABAergic presynaptic inhibitory neurotransmission [168]. Crosby and colleagues (2011) [169] demonstrated that the ECS may both promote long-term depression and gate the long-term potentiation of GABAergic synapses in the dorsomedial hypothalamus, which seems to be dependent on nitric oxide signaling and satiety state [169]. The biphasic effect of endocannabinoids in relation to feeding behavior was also detected in the ventromedial hypothalamus. It was shown that low-to-moderate doses of CB1 receptor agonists increased food intake [170], but higher levels exerted anorectic effects [171]. As for the paraventricular nucleus, the activation of the CB1 receptors located in this brain area causes the inhibition of serotonin release with reduced serotonin neurotransmission via the 5-HT1A and 5-HT1B serotonin receptors, as well as the stimulation of GABA release, which, as a consequence, results in increased food intake [172]. Endocannabinoid synthesis and release in the paraventricular nucleus may be inhibited by leptin, which enhances the release of glutamate [173].

In the mesolimbic system, CB1 receptors have an impact on dopaminergic transmission. The stimulation of CB1 receptors in the ventral tegmental area–nucleus accumbens circuit and ventral tegmental area–medial prefrontal cortex is highly important for the modulation of hedonic feeding behaviors. In the ventral tegmental area, CB1 receptors are expressed on both presynaptic GABAergic and glutamatergic terminals. The activation of CB1 receptors on GABAergic inputs diminishes the release of GABA, which results in the increased release of dopamine in the nucleus accumbens and hedonic feeding behaviors. On the other hand, the activation of CB1 receptors on glutamatergic inputs reduces the release of glutamate, which results in the suppression of the excessive stimulation of dopaminergic neurons [174,175]. In the nucleus accumbens, CB1 receptors are located on presynaptic glutamatergic terminals. The excitation of these receptors diminishes the release of glutamate onto spiny projection neurons, which results in prolonged and intensified dopaminergic signals related to reward [176,177]. Furthermore, dopamine release in the nucleus accumbens may induce the release of endocannabinoids, which augments the activation of CB1 receptors and thus maintains the cycle of reward-driven eating. The chronic stimulation of the ECS results in excessive dopamine release, which causes the adaptation of reward sensitivity. As a consequence, in order to achieve the same hedonic response, larger amounts of food are required, which is associated with binge-eating behaviors or overeating [178]. Interestingly, according to Mazier and colleagues (2015) [37], AEA and 2-AG may have distinct roles in the modulation of eating behavior. AEA, whose levels peak in the plasma of normal-weight subjects before meals and decrease just after meals, may act as a physiological initiator of eating [19]. As for 2-AG, its plasma level does not correlate with physiological need/food deprivation but with the availability of palatable food [20]. Monteleone and colleagues (2012) [20] reported that the plasma concentrations of 2-AG were considerably elevated before and after the ingestion of palatable food by healthy volunteers. This pre-meal release of 2-AG and post-meal sustained elevation of its peripheral levels indicate that 2-AG is involved in both anticipation and reward (i.e., hedonic eating behaviors in humans).

The ECS also has an impact on the hippocampus and olfactory neuronal circuits. Both of them regulate feeding behavior [179,180]. The olfactory bulb is responsible for odor-driven hedonic eating [181], and the hippocampus is involved in food-associated memories [182]. Soria-Gomez et al. (2014) [183] found that food deprivation elevates endocannabinoid levels in the olfactory bulb and stimulates CB1 receptors located on the axon terminals of the olfactory cortex, which, in turn, causes intensified odor detection and increased food intake when animals are re-exposed to food.

Interestingly, recent studies carried out by Martin-Garcia et al. (2025) [184] demonstrated that evolutionarily, the CaMKII neuron subpopulation is instrumental in feeding behavior under physiological conditions. It is responsible for shifting energy balance to energy accumulation by promoting food seeking and overfeeding, reducing the expenditure of energy, and facilitating lipid storage. The expression of CB1 receptors on these neurons is sufficient to develop obesity and other eating disorders under hypercaloric conditions.

Whereas the central ECS is involved in the modulation of feeding behavior in response to hunger, emotional aspects, and reward, the peripheral ECS primarily expressed in the skeletal muscles, gastrointestinal tract, liver, pancreas, and adipose tissue [14] is involved in metabolic and physiological processes related to eating [185]. Generally, the activation of peripheral CB1 receptors promotes appetite, energy storage, and preservation [22]. CB1 receptors are localized on the peripheral terminals of sensory neurons [186] and on the peripheral terminals of the parasympathetic and sympathetic nervous systems [187,188]. The induction of CB1 receptors within the gastrointestinal tract results in an increased sweet sensitivity in the oral cavity [189], decreased gastric secretion and acetylcholine release, elevated gastric release of ghrelin, delayed gastric emptying [190,191,192], diminished secretion cholecystokinin, slowed motility of the gastrointestinal tract [193,194], and modulated gastric vagal afferent mechanosensitivity [195]. Avalos et al. (2020) [196] reported that mice with the deletion of CB1 receptors in the intestinal epithelium did not present a preference for a Western-style diet (high-fat/sucrose), which is largely preferred by their wild-type counterparts. The stimulation of CB1 receptors present on adipocytes is associated with reduced lipolysis, enhanced fat storage, and hyperleptinemia, as well as elevated expression levels of leptin and diminished expression levels of adiponectin in adipose tissue, whereas their inhibition results in stronger energy expenditure [114,197]. De Azua and colleagues (2017) [114] demonstrated that the deletion of the *CB1* gene in adipocytes protected adult mice from diet-induced obesity and obesity-related metabolic anomalies. The activation of CB1 receptors located in hepatocytes leads to increased lipogenesis and fatty acid synthesis, dyslipidemia, gluconeogenesis, and hepatic insulin resistance. In experiments by Osei-Hyiaman et al. (2008) [198], the selective deletion of hepatic CB1 receptors reduced the degree of hyperglycemia, dyslipidemia, and insulin and leptin resistance in mice with diet-induced obesity [198]. The stimulation of CB1 receptors in the skeletal muscles has an impact on peripheral insulin resistance [197], whereas the ablation of muscle CB1 receptors prevents diet-induced insulin resistance and increases whole-body muscle energy expenditure [199]. Eventually, CB1 receptors expressed in the β-cells of pancreatic islets modulate both β-cell proliferation and insulin receptor signaling. The activation of these receptors has a negative impact on insulin action and leads to β-cell death, whereas a pharmacological blockade or genetic deficiency of CB1 receptors improves insulin performance and glucose responsiveness [197,200]. Tam et al. (2017) [201], Senin et al. (2013) [192], and Argueta et al. (2019) [202] gave evidence that the peripheral blockage of CB1 receptors increases leptin sensitivity, reduces ghrelin secretion, and induces the secretion of cholecystokinin, respectively. Additionally, most probably, the modification of the microbiome by the peripheral ECS contributes to the modulation of body weight by CB1 receptors [203]. However, as was observed by Permyakova et al. (2023) [204], the dysregulation of peripheral endocannabinoid transmission in obesity is not limited to the alimentary system and skeletal muscles. Obese patients with kidney lesions presented higher kidney levels of AEA, reduced expression levels of CB1 receptors, and increased activity of enzymes participating in the synthesis and degradation of endocannabinoids. The peripheral ECS interacts with the CNS, affecting hunger and satiety signals [185].

**Figure 1:**
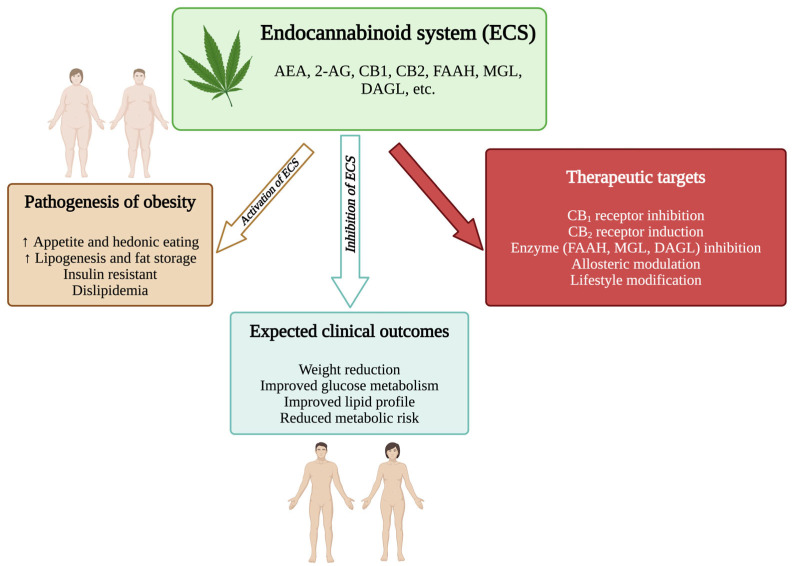
The endocannabinoid system in obesity. 2-AG, 2-arachidonoylglycerol; AEA, anandamide; CB1, cannabinoid receptor type 1; CB2, cannabinoid receptor type 2; DAGL, diacylglycerol lipase; ECS, endocannabinoid; MGL, monoacylglycerol lipase. (Figure generated using BioRender.com).

**Figure 2:**
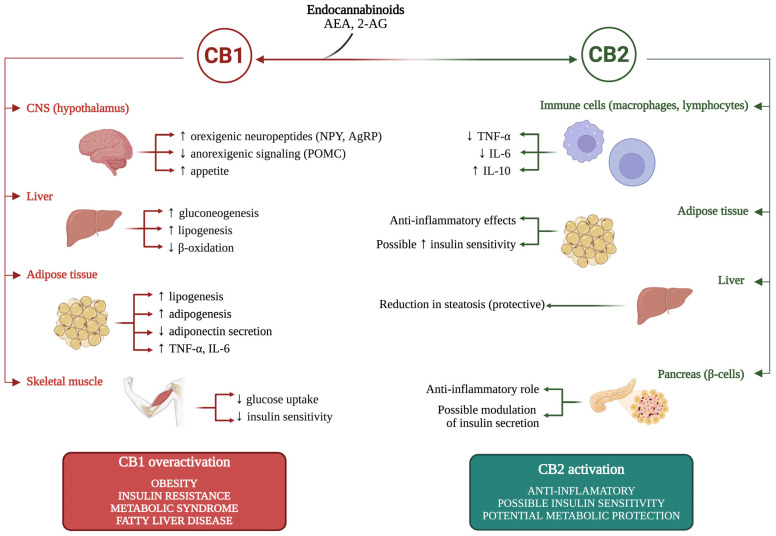
Schematic representation of CB1 and CB2 receptor-mediated pathways and their links to obesity. 2-AG, 2-arachidonoylglycerol; AEA, anandamide; AgRP; agouti-related peptide; CB1, cannabinoid receptor type 1; CB2, cannabinoid receptor type 2; CNS, central nervous system; IL-6, interleukin 6; IL-10, interleukin 10; NPY, neuropeptide Y; POMC, pro-opiomelanocortin; TNF-α, tumor necrosis factor alpha. (Figure generated using BioRender.com).

A graphical summary of ECS involvement in obesity is presented in Figure 1 and Figure 2.

## 4. Cannabinoid-Based Treatments in Obesity

Since both the peripheral and central ECSs have an impact on feeding behaviors, scientists and clinicians are working together on cannabinoid-based treatments for eating-related diseases, including obesity. Molecules that either interact with CB (mainly CB1) receptors or substances that modulate the activity of enzymes that are responsible for the metabolic turnover of endocannabinoids are primarily taken into consideration. It has been shown that the modulation of central CB1 receptors can affect neuronal and circuit excitability and influence reward-based eating and appetite, while modifications in signaling dependent on peripheral CB1 receptors may influence the secretion of key hormones regulating hunger and satiety or stimulate lipogenesis [52]. Unfortunately, the first option is related to the occurrence of central side effects (i.e., reduced joy, low mood, anxiety, depression), whereas the second one has a lower burden of mental adverse reactions [205].

At the beginning of the XXI century, particular attention was directed towards antagonists/inverse antagonists of CB1 receptors in the management of body weight [44,206,207]. One of the anorectic anti-obesity cannabinoid-based drugs was rimonabant, an inverse agonist of the CB1 receptor. Approved in 38 countries, including EU, it was indicated as an adjunct to diet and exercise for the treatment of obese or overweight patients with associated risk factors, such as type 2 diabetes or dyslipidemia. In participants of clinical trials, rimonabant significantly reduced body weight and waist circumference and improved the levels of high-density lipoprotein cholesterol (HDL-C), triglycerides, and glycated hemoglobin (HbA1c). However, after 3 years of being on the pharmaceutical market, it was withdrawn due to psychiatric adverse reactions, including an increased risk of suicidality. As a consequence of this decision, CB1 receptor inverse antagonists (i.e., taranabant, otenabant, ibipinabant, surinabant) were withdrawn by pharmaceutical companies from clinical trials [208]. However, several years later, tests on CB1 receptor ligands started again. This time, scientists mainly focused on peripherally acting molecules, with a reduced ability to pass the blood–brain barrier. Such a proceeding was understandable, particularly in view of the fact that the expression of peripheral CB1 receptors is significantly elevated in obese and diabetic patients, while in healthy people, it is very low [116,209,210]. Both inverse agonists and neutral antagonists have been evaluated. The CB1 receptor possesses constitutive activity in the absence of any ligand. According to Meye et al. (2013) [205], neutral antagonists could be a safer alternative to inverse agonists (though less effective in control of appetite), since they inhibit CB1 receptors without suppressing their basal activity. Among these “second-generation” peripherally restricted CB1 receptor ligands, often based on a rimonabant template or, alternatively, on an ibipinabant scaffold, URB447 (a mixed CB1 receptor antagonist/CB2 receptor agonist), AM6545, BPRCB1184, VD60, THCV, LH-21, AM4113, NESS06SM, SM-11, PIMSR (CB1 receptor antagonists), JD5037, JD5006, BPR697, TM38837, AM251, AJ5012, AJ5018, MRI-1867, BPR0912, TXX-522, and ENP11 (CB1 receptor inverse agonists) were tested in preclinical studies, as well as 2,3-diarylpyrroles, substituted hydantoins, amide derivatives of the 1,5-diaryl-3-carboxylic acid, benzhydrylpiperazines, 6-alkoxy-5-aryl-3-pyridinecarboxamides, 6-benzhydryl-4-aminoquinolin-2-ones, cinnoline derivatives, aryl alkynyl-thiophene compounds, pyrazolyl compounds, 1,1-dioxo-thiomorpholino compounds, 1,5-diaryl-4,5-dihydro-1H-pyrazole-3-carboxamid compounds, and others. Generally, they increase lipid mobilization, decrease triglyceride storage, have a negative impact on glucose production, and improve glucose metabolism, the secretion of islet hormones, and/or the secretion of adipokines. As a consequence of their action, decreased food intake and body weight and improved insulin tolerance and lipid profile are anticipated (see [197,211,212]). Particular attention was given to AM6545, JD5037, AM4113, AM251, BAC, and TM38837. It was demonstrated that chronic treatment with the first two of the above-mentioned compounds was able to reduce caloric intake, body weight, and adiposity and improve glucose intolerance, insulin resistance, and dyslipidemia in mice with diet-induced obesity. Neither AM6545 nor JD5037 negatively affected the brain of the treated animals [213,214]. The anti-obesity effect of JD5037 was attributed to the reversal of leptin resistance [214]. AM4113, another neutral antagonist of CB1 receptors (the pyrazole-based one), transiently diminished food intake in rats and reduced their body weight gain in a dose-dependent manner when given intraperitoneally or orally [215], though in experiments by Sink and colleagues (2009) [216], its oral bioavailability was low [216]. AM251, which is structurally similar to rimonabant, induces hypophagia, promotes weight loss and energy expenditure, and decreases adiposity in obese rodents [217,218,219]. BAC, a new thiazolidine derivative with pleiotropic antagonistic activity towards cannabinoid CB1 receptors, reduced obesity and improved both glucose and lipid parameters in mice with diet-induced diabesity. The compound did not exert any detrimental effects on the neurobehavior of animals [218,220]. As for TM38837, which exerts similar effects in rodent obesity models as rimonabant [220], it entered clinical trials. In a double-blind, randomized, placebo-controlled, crossover study with healthy volunteers, it was demonstrated that this compound at a dose of 100 mg did not penetrate the brain [221]. However, in an experiment by Micale and colleagues (2019) [222], TM388837 at a dose of 100 mg/kg given per os induced fear-promoting effects in mice. Still, such a dose was at least one order of magnitude lower than the anxiogenic dose of rimonabant. Recently, in a multicenter, randomized, double-blind, placebo-controlled clinical trial with adult participants presenting features of metabolic syndrome, monlunabant (INV-202/MRI-1891), a novel CB1 receptor inverse agonist, caused a reduction in waist circumference and body mass index when given orally at a daily dose of 25 mg for 28 days. The tested agent was well-tolerated with adverse reactions mainly related to the gastrointestinal tract. It also improved lipid parameters, such as total cholesterol, triglycerides, LDL-C, and non-HDL-C [223]. In experiments in mice, INV-202 was effective in obese asthma. The agent caused weight loss and improved airway parameters [224]. Surprisingly, quite recently, Matheson and colleagues (2025) [225], in a randomized, double-blind, placebo-controlled pilot clinical trial with obese adults, tried to check whether nabilone, a partial CB1 receptor agonist, would reduce the body weight of the participants. Even though a significant intervention effect on both body weight and body mass index was detected (at a dose of 2 mg/day), these outcomes cannot be taken as reliable since the study was terminated early and only 8 out of 18 subjects completed the study due to the poor tolerability of nabilone. In fact, the agonists and partial agonists of CB1 receptors had been studied before in the context of obesity treatment, however, with conflicting results (see [211]). For example, the outcomes of the latest experiments in mice carried out by Eitan et al. (2023; 2024) [226,227] suggested that either the prolonged oral intake of Δ9-tetrahydrocannabinol or consumption of Δ9-tetrahydrocannabinol-enriched cannabis oil may reduce diet-induced weight gain and improve glucose tolerance. A randomized, placebo-controlled clinical trial evaluating the effects of a once-a-day oral intake of cannabis for weight loss in obese individuals is going to be started this year (NCT06137365) [226,227].

Targeting enzymes that take part in the biosynthesis or/and degradation of endocannabinoids in order to reduce their levels is another promising research direction. Scientists have predominantly focused on the enzymes involved in the metabolic turnover of 2-AG, since studies on *N*-arachidonoyl phosphatidylethanolamine phospholipase D and fatty acid amide hydrolase brought ambiguous results. It should be mentioned here that fatty acid amide hydrolase degrades pro-appetitive and anti-appetitive compounds. Apart from endocannabinoids, oleoylethanolamine and palmitoylethanoamine belong to substrates of the mentioned enzyme. Both oleoylethanolamine and palmitoylethanoamine have an impact on satiety and metabolism, with effects opposite to the ones observed for 2-AG and AEA. Thus, the observed outcome of the modulation of fatty acid amide hydrolase activity may be influenced not only by endocannabinoids but also by other endogenous substances [228,229]. Gao et al. (2010) [230] and Powell et al. (2015) [231] demonstrated that mice with the deletion of the gene encoding diacylglycerol lipase-α presented significantly lower levels of 2-AG in the brain and were leaner when compared to wild-type animals; they had low fasting insulin, triglyceride, and total cholesterol levels. Furthermore, their body weight, biochemical parameters, and behavior were comparable to parameters/responses observed in mice with the deletion of the *CNR1* gene. Even though mice with the deletion of the gene encoding diacylglycerol lipase-β had relatively lower levels of 2-AG in the brain than their wild-type counterparts, their body weight was not different [230,231]. Bisogno et al. (2013) [232] demonstrated that O-7460, a new fluorophosphonate, which acts as an inhibitor of diacylglycerol lipase, when given by an intraperitoneal injection, diminished the intake of a high-fat diet and reduced the body weight of the exposed mice. Tetrahydrolipstatin, another inhibitor of diacylglycerol lipase, administered into the lateral hypothalamus, diminished food intake in rats [233]. If the inhibition of the enzyme that takes part in the biosynthesis of 2-AG results in reduced body weight and/or food intake in rodents, the stimulation of enzymes that degrade this endocannabinoid should lead to similar effects. However, it seems that the outcomes of the overexpression of monoacylglycerol lipase are dependent on its location. Transgenic mice with an upregulated enzyme in the small intestine presented an obese phenotype (i.e., hyperphagia, elevated body weight gain, and body fat mass, reduced energy expenditure) and lower levels of 2-AG in the small intestine [143]. On the other hand, transgenic mice with the upregulated enzyme in the forebrain, had lower levels of 2-AG in this brain region, but they were lean and resistant to diet-induced obesity [144]. Furthermore, monoglyceride lipase-deficient mice displayed increased levels of 2-AG but were characterized by normal food intake, fat mass, and energy expenditure. Nevertheless, when given a high-fat diet, they had better glucose tolerance and insulin sensitivity than their wild-type counterparts [234].

The outcomes from several experiments carried out in rodents with diet-induced obesity demonstrated that reduced food intake and body weight may be obtained by an enhanced stimulation of CB2 receptors, e.g., via the administration of their agonists [125], though the findings are not consistent across studies [235]. However, the simultaneous antagonism of CB1 receptors with the partial agonism of CB2 receptors seems to be a promising therapeutic option for the management of obesity. Such biological activity involves tetrahydrocannabivarin, which belongs to the cannabinoids derived from *Cannabis sativa*. Preclinical data indicate that this substance suppresses appetite and food intake, improves glucose metabolism, augments insulin sensitivity, prevents excessive energy storage, and reduces systemic inflammation (for a review, see [236,237]). Furthermore, it had a favorable safety profile (without psychoactive adverse reactions) in patients with 2 diabetes [238] and healthy individuals [239]. As was reported by Tudge and colleagues (2014) [239], tetrahydrocannabivarin increased neural responses to rewarding and aversive stimuli (i.e., sight and/or flavor of chocolate versus picture of moldy strawberries and/or a less pleasant strawberry taste). Furthermore, in a randomized placebo-controlled trial with the participation of obese patients, conducted by Smith (2025) [240], the administration of mucoadhesive oral strips containing tetrahydrocannabivarin and cannabidiol (8 mg/10 mg or 16 mg/20 mg) for 90 days was associated with significant weight loss. The treatment was well-tolerated in both tested doses [240]. Another mixed CB1 antagonist/CB2 agonist, URB447, reduced total food consumption and inhibited body weight gain in mice with an efficacy similar to the one observed for rimonabant but without exerting central effects. According to the results obtained by LoVerme and colleagues (2009) [241], this pyrrole-based cannabimimetic does not penetrate the brain.

The allosteric negative modulation of CB1 receptors is a relatively new idea in research on cannabinoid-based treatments in obesity. Apart from orthosteric binding sites, CB1 receptors most likely also have allosteric binding sites. Agents that interact with allosteric binding sites induce conformational changes within the receptor and influence the activity of the receptor orthosteric ligands. When so-called positive allosteric modulation supports ligand binding, negative allosteric modulation decreases the specific binding of the orthosteric agonists/antagonists. One of these modulatory agents is PSNCBAM-1. It seems that this compound has a positive impact on the binding of orthosteric CB1 receptor agonists, but it behaves as an antagonist of agonist-induced responses. As for the orthosteric antagonists/inverse agonists of CB1 receptors, PSNCBAM-1 moderately decreases their binding. In preclinical studies, PSNCBAM-1 via the allosteric antagonism of CB1 receptors decreased food intake and body weight in rats [242]. The research conducted by Leone (2018) [243] found that RVD-hemopressin(α), which serves as an allosteric modulator of CB1 and CB2 receptors, inhibited the food intake of rats fed with a highly palatable cafeteria-style diet or standard diet, but it did not influence the body weight of the tested animals.

Currently, scientists are working on “the third generation” of CB1 receptor antagonists, which are peripherally restricted and have two targets. Hybrid agents block CB1 receptors and may either stimulate or inhibit the second target. In the case of hybrid CB1 receptor/inducible nitric oxide synthase (iNOS) antagonists, iNOS as a secondary target is also inhibited. The orally available hybrid inhibitors of CB1 receptors and iNOS, MRI-1569 and MRI-1867, which do not penetrate the brain, have shown promising anti-obesity activity related to reduced body weight and food intake in mice with diet-induced obesity [197,244]. Recently, Bhattacharjee and colleagues (2025) [245] evaluated two novel orally available tetrahydropyridazine-based molecules, i.e., PB19A and PB95, that are peripherally restricted CB1 receptor antagonists with iNOS inhibitor properties. Surprisingly, it was found that the compounds had very modest effects in terms of the reduction in body weight and food intake in dietary-induced obese mice [245]. Another hybrid molecule, VCE-004.8, that stimulates both CB2 and PPARγ receptors diminished body weight gain, total fat mass, and adipocyte volume in mice fed with a high-fat diet. Apart from that, it improved glucose tolerance and ameliorated metabolic parameters associated with obesity, i.e., reduced the levels of triglycerides and leptin and increased the levels of adiponectin and incretins [246]. Furthermore, in experiments by Cotrim et al. (2012) [247] and Decara et al. (2015) [248], dual PPAR-α/CB1 receptor ligands (i.e., compound 7 and OLHHA) exerted a hypophagic effect in wild-type Wistar rats and lean Zucker rats, respectively. Additionally, OLHHA reduced the body weight gain in lean (but not obese) Zucker rats. Stefanucci and colleagues (2018) [249] presented novel hybrids of rimonabant and Fubinaca family agents. Their proposed mechanism of action would be via the selective antagonism or inverse agonism of CB1 receptors. However, neither the toxicological nor pharmacokinetic properties of these compounds have been studied yet [249]. Research on novel dual inhibitors of alpha/beta-hydrolase domain-containing 6 and monoacylglycerol lipase, diacylglycerol lipase alpha, and fatty acid amide hydrolase has also been carried out. Alpha/beta-hydrolase domain-containing 6 is an enzyme that contributes to the postsynaptic degradation of 2-AG [250].

An overview of therapeutic strategies targeting the ECS is provided in Table 3.

### New Perspectives in the Cannabinoid-Based Treatment of Obesity

The Ozempic case proved that millions of obese patients are waiting for a drug effective in weight management, some of them for health reasons, others for esthetic reasons. However, these drugs must have a positive safety profile and be free from serious side effects. Nobody wants the same scenario as was seen with rimonabant, the drug that had raised false hopes. Currently, no CB receptor ligand-based anti-obesity/weight-reducing treatment is available, but various CB receptor-acting agents are being tested, mainly in preclinical studies, including inverse agonists or neutral antagonists of the CB1 receptor, agonists of CB2 receptors, compounds influencing the functioning of the main enzymes of the ECS, allosteric modulators of CB1 receptors, and hybrid compounds. Some of them exert comparable effects to rimonabant in experiments in rodents, while in silico studies suggest their more beneficial safety profile. A lot of them also have the potential to be useful in the management of diabetes, dyslipidemia, or metabolic disorder. However, many studies concerning the role of the agents interfering with components of the ECS demonstrate either conflicting results or provide ambiguous outcomes (particularly related to the activity of CB2 receptor ligands or enzymes involved in biosynthesis and degradation of endocannabinoids, which are not selective for 2-AG or AEA), which highlights a necessity to carry out further research. Additionally, more research is needed to define the pharmacokinetics and understand the mechanisms of the anti-obesity action of novel CB receptor-acting compounds.

Though a great percentage of preclinical studies have been carried out in animal models of diet-induced obesity, i.e., DIO, which is more similar to obesity in people than genetically engineered obesity, the findings from such experiments should be confirmed in the human population. However, before introducing an ECS-focused agent into clinical trials, its safety profile must be determined reliably. Safety in terms of the CNS is still a big concern for scientists. They have been working on the optimalization of the physicochemical properties of the above-mentioned agents to restrict their activity to the maximum extent to the peripheral ECS. Preferably, they should not penetrate the brain, or their ability to pass the blood–brain barrier should be negligible. Furthermore, ideally, they should have a short elimination half-life in order to not accumulate within the body. On the other hand, characteristics that are important for peripheral activity (e.g., reduced permeability via biological membranes or higher polarity) may at the same time limit oral availability, which is a major drawback for an anti-obesity drug. Obese people usually suffer from other chronic diseases and take different drugs. Therefore, it is crucial to exclude the risk of potential drug–drug and drug–disease interactions. And finally, anti-obesity therapy should be longer. So, it is important to investigate the safety of long-term use.

Amongst the proposed novel options for weight reduction by acting on the ECS, some non-traditional methods are also mentioned, including dietary interventions, exercise, and hypoxia training. Kim et al. (2013) [251] presented the idea “fat to treat fat”, according to which an increased consumption of omega-3 polyunsaturated fatty acids with a reduced intake of omega-6 polyunsaturated fatty acids (i.e., precursors of endocannabinoids) lowers the plasma levels of endocannabinoids [252,253] and, as a consequence, may prevent or treat excessive body weight. The same conclusions were drawn by Sierra-Ruelas et al. (2023) [254], who, having evaluated the relationship between the *FAAH* Pro129Thr variant and metabolic phenotypes in the Mexican population, suggested that a low dietary intake of polyunsaturated fatty acids, which are precursors of endocannabinoids, may partly prevent the development of an unfavorable overweight/obesity-related lipid profile. The outcomes of a recent 12-week randomized controlled intervention trial [255,256] showed that both a low-nutrient-quality diet (high in saturated fats, unsaturated fats, and fructose) and a high-nutrient-quality energy-restricted diet (enriched in monounsaturated fatty acids, polyunsaturated fatty acids, soy protein, and fiber) significantly diminished body weight, body mass index, and waist circumference, as well as improving the glucose and triglyceride profile in people with abdominal obesity when compared with participants consuming a habitual diet. Furthermore, these changes in metabolic parameters, body weight, and body mass index were more beneficial in individuals on the high-nutrient-quality diet. Following such a diet (containing 1224 mg of omega-3-fatty acids) prevented a decrease in the plasma and adipose tissue levels of the docosahexaenoic acid (DHA)-derived endocannabinoid-like compound dehydroepiandrosterone (DHEA) and increased the expression of the gene encoding diacylglycerol lipase α. Also, an inulin-enriched diet seems to be a promising intervention for both the prevention and treatment of obesity. Alptekin and colleagues (2024) [257] observed that inulin prevented body weight gain induced by a high-fat diet and diminished food intake in Wistar rats. The authors found that these effects were at least partially mediated by the ECS since the tested rats exhibited suppressed *CNR1* expression in the prefrontal cortex as well as reduced prefrontal levels of AEA and 2-AG [257]. Inulin also promoted weight loss in human subjects [258]. The outcomes of the recent experiments carried out by Cocci et al. (2021) [259] indicated tart cherry supplements as a promising dietary intervention that could be able to downregulate the expression of CB1 receptors in the adipose tissue of rats with obesity induced by a high-fat animal feed. Interestingly, the maternal supplementation of fish oil during pregnancy decreased the activation of liver ECS signaling in the newborn rats of dams receiving a high-fat diet. The effect was independent of sex. The authors detected reduced liver contents of CB receptors (CB1 and CB2), AEA, and 2-AG [71].

Several studies indicated that hypoxia training is a safe procedure that reverses the overactivation of the ECS and has beneficial effects on weight loss and weight regain in both obese adolescents and adults [260,261,262]. An additional non-pharmacological strategy should be exercise [263,264]. Elisei and colleagues (2023) [265], who compiled data on the association between the ECS and aerobic/resistance physical training, concluded that exercise could modulate the imbalance of this system in obesity. Park and Watkins (2022) [266] also focused their attention on the relationship between dietary polyunsaturated fatty acids, exercise, and the functionality of the ESC.

Finally, Pucci et al. (2019) [267] paid attention to the possibilities of early diagnosis and a preventive approach. It seems that an altered regulation of the *CNR1* gene occurs at the beginning of the development of the obese phenotype, and it was correlated with hypomethylation at the *CNR1* gene [267]. These findings were in accordance with the results obtained by He et al. (2019) [268], who investigated the association between the level of DNA methylation in obesity-related genes and body mass index in adolescents. When diagnosed early, diet and physical activity might prevent obesity in later stages of life.

## 5. Conclusions

The quest for efficacious and safe cannabinoid-based therapies for obesity is continuing. Despite the demonstrated efficacy of numerous CB receptor ligands and enzyme modulators in preclinical studies, their clinical translation is impeded by safety concerns, pharmacokinetic limitations, and a paucity of long-term data. Peripherally restricted CB1 antagonists, hybrid compounds, and allosteric modulators have the potential to overcome some of these challenges; however, careful evaluation in humans is imperative. In addition to pharmacological approaches, dietary strategies, exercise, and hypoxia training have emerged as complementary tools capable of modulating the ECS and improving metabolic outcomes. Furthermore, early diagnosis and preventive measures targeting ECS-related genetic and epigenetic changes may offer additional opportunities to reduce the burden of obesity. Collectively, these findings underscore the therapeutic potential of the ECS while emphasizing the necessity for meticulous translational research and prolonged safety studies before the efficacious implementation of novel interventions in clinical practice can be assured.

Taken together, the growing body of evidence suggests that targeting the ECS, either pharmacologically or through lifestyle interventions, may open a new chapter in the prevention and treatment of obesity. Ultimately, the ECS represents a promising but still challenging target, and successful translation into clinical practice will depend on efficacy with long-term safety.

## Figures and Tables

**Table 1:** Increased body weight (HP:0004324) according to the Human Type Ontology.

Term		Definition	ID
Large for gestational age		This applies to babies whose birth weight lies above the 90th percentile for that gestational age.	HP:0001520
Obesity		Accumulation of substantial excess body fat.	HP:0001513
	Abdominal obesity	Excessive fat around the stomach and abdomen.	HP:0012743
	Class III obesity	Obesity with a body mass index of 40 kg per square meter or higher.	HP:0025501
	Class II obesity	Obesity with a body mass index of 35 to 39.9 kg per square meter.	HP:0025500
	Class I obesity	Obesity with a body mass index of 30 to 34.9 kg per square meter.	HP:0025499
	Truncal obesity	Obesity located preferentially in the trunk of the body as opposed to the extremities.	HP:0001956
Overweight		Increased body weight with a body mass index of 25–29.9 kg per square meter.	HP:0025502

**Table 2:** Cryo-EM structure of human cannabinoid receptors CB1 and CB2 in complex with signaling proteins.

Receptor	Ligand (Agonist)	Complexed Protein	Resolution	PDB ID	Structure	Ref.
CB1	MDMB-Fubinaca	Gi	3.0 Å	6N4B	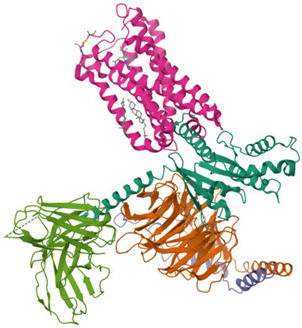 PDB doi.org/10.2210/pdb6N4B/pdb	[82]
	AMG315	Gi	2.80 Å	8GHV	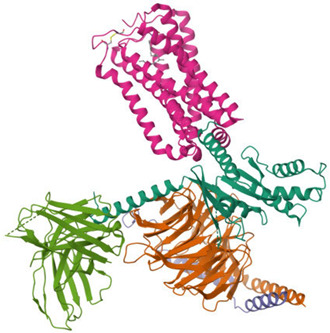 PDB doi.org/10.2210/pdb8GHV/pdb	[90]
	ZCZ011	Gi	3.36 Å	7WV9	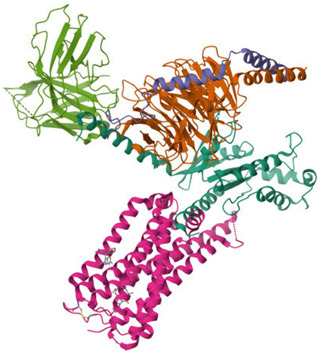 PDB doi.org/10.2210/pdb7WV9/pdb	[96]
CB2	WIN 55,212-2	Gi	3.2 Å	6PT0	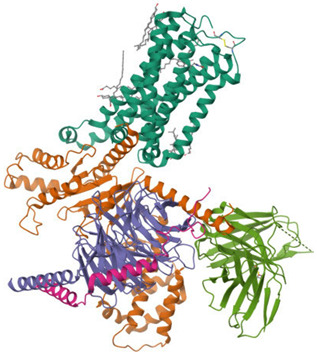 PDB doi.org/10.2210/pdb6PT0/pdb	[94]
	LEI-102	Gi	2.98 Å	8GUT	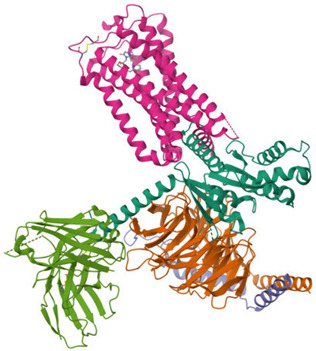 PDB doi.org/10.2210/pdb8gut/pdb	[97]

**Table 3:** Overview of strategies targeting the ECS for the treatment of obesity.

Strategy	Mechanism of action	Example compounds	Research status	Advantages	Limitations
CB1 antagonists/inverse agonists	Appetite suppression, reduction of lipogenesis	AM6545, JD5037, TM38837, Rimonabant	Clinical/preclinical	Strong anorectic effect	Psychiatric adverse events (if crossing the CNS)
CB2 agonists	Anti-inflammatory effect, improved metabolism	Tetrahydrocannabivarin, URB447	Preclinical	Lack of neuropsychiatric effects	Inconsistent findings
Allosteric CB1 modulators	Selective regulation of receptor activity	PSNCBAM-1, RVD-hemopressin	Preclinical	Potentially safer profile	Early research stage
Enzyme inhibitors (FAAH, MGL, DAGL)	Modulation of endocannabinoid levels	O-7460, tetrahydrolipstatin	Preclinical	Endogenous regulation	Mixed/ambiguous results, lack of selectivity
Hybrid compounds (dual target)	CB1 antagonism with additional target (e.g., PPARγ, iNOS)	VCE-004.8, MRI-1867	Preclinical	Synergistic action	No clinical data
Non-pharmacological interventions	ECS modulation via diet and lifestyle	Mediterranean diet, omega-3, inulin, exercise, hypoxia training	Experimental/clinical	Safe, low-cost	Less potent than pharmacotherapy

CB1, cannabinoid receptor type 1; CB2, cannabinoid receptor type 2; CNS, central nervous system; DAGL, diacylglycerol lipase; ECS, endocannabinoid system; FAAH, fatty acid amide hydrolase; iNOS, inducible nitric oxide synthase; MGL, monoacylglycerol lipase; PPARγ, peroxisome proliferator-activated receptor gamma

## Data Availability

No new data were created or analyzed in this study. Data sharing is not applicable to this article.

## Abbreviations

The following abbreviations are used in this manuscript:

2-AG	2-arachidonoylglycerol
5-HT	Serotonin receptor
7TM	Seven-transmembrane
AEA	*N*-arachidonoyl-ethanolamine, anandamide
C-term	Carboxyl-terminal domain
CB1	Cannabinoid receptor type 1
CB2	Cannabinoid receptor type 2
*CNR1*	Gene encoding cannabinoid receptor type 1
*CNR2*	Gene encoding cannabinoid receptor type 2
CNS	Central nervous system
DAGL	Diacylglycerol lipase
DHA	Docosahexaenoic acid
DHEA	Dehydroepiandrosterone
ECL	Extracellular loop
ECS	Endocannabinoid system
FAAH	Fatty acid amide hydrolase
GABA	Gamma-aminobutyric acid
GPR	G protein-coupled receptor
HbA1c	Glycated hemoglobin
HDL-C	High-density lipoprotein cholesterol
ICL	Intracellular loop
iNOS	Inducible nitric oxide synthase
LDL-C	Low-density lipoprotein cholesterol
MAPK	Mitogen-activated protein kinase
MGL	Monoacylglycerol lipase
N-term	Amino-terminal domain
NAPE-PLD	*N*-acyl phosphatidylethanolamine phospholipase D
PPAR	Peroxisome proliferator-activated receptor
ROCK	Rho-associated coiled-coil kinase
TRPV1	Transient receptor potential vanilloid 1
